# A narrative method for consciousness research

**DOI:** 10.3389/fnhum.2013.00739

**Published:** 2013-11-08

**Authors:** José-Luis Díaz

**Affiliations:** Department of History and Philosophy of Medicine, Faculty of Medicine, National Autonomous University of MexicoMexico City, Mexico

**Keywords:** narrative method, consciousness research, interior monolog, phenomenological texts, conscious process models, dynamic system model, heterophenomenology, neurophenomenology

## Abstract

Some types of first-person narrations of mental processes that constitute phenomenological accounts and texts, such as internal monolog statements, epitomize the best expressions and representations of human consciousness available and therefore may be used to model phenomenological streams of consciousness. The type of autonomous monolog in which an author or narrator declares actual mental processes in a think aloud manner seems particularly suitable for modeling streams of consciousness. A narrative method to extract and depict conscious processes, operations, contents, and states from an acceptable phenomenological text would require three subsequent steps: operational criteria for producing and/or selecting a phenomenological text, a system for detecting text items that are indicative of conscious contents and processes, and a procedure for representing such items in formal dynamic system devices such as Petri nets. The requirements and restrictions of each of these steps are presented, analyzed, and applied to phenomenological texts in the following manner: (1) the relevance of introspective language and narrative analyses to consciousness research and the idea that specific narratives are of paramount interest for such investigation is justified; (2) some of the obstacles and constraints to attain plausible consciousness inferences from narrative texts and the methodological requirements to extract and depict items relevant to consciousness contents and operations from a suitable phenomenological text are examined; (3) a preliminary exercise of the proposed method is used to analyze and chart a classical interior monolog excerpted from James Joyce’s *Ulysses,* a masterpiece of the stream-of-consciousness literary technique and, finally, (4) an inter-subjective evaluation for inter-observer agreement of mental attributions of another phenomenological text (an excerpt from the *Intimate Journal* of Miguel de Unamuno) is presented using some mathematical tools.

“Let us record the atoms as they fall upon the mind in the order in which they fall, let us trace the pattern, however, discontinued and incoherent in appearance, with which each sight or incident scores upon the consciousness.”

Woolf (1925; The Common Reader)

## INTRODUCTION: SUBJECTIVE NARRATIVES AND CONSCIOUSNESS RESEARCH

A fundamental methodological assumption of neurophenomenology is that subjective verbal reports expressed by people about what goes on in their minds is a critical and necessary source to understand conscious processing and its neural and other bodily correlates (Varela, 1996). Moreover, such reports may be construed as to provide a pragmatic evidence of the embodied character of mental life in terms of its neurobehavioral, sensorimotor, expressive, communicative, and enactive nature. The phenomenological approach to analyze other people’s consciousness was extensively employed after the 1920s by European psychiatrists of the stature of Karl Jaspers, Joseph Minkowski, or Ludwig Binswanger, in their attempt to apply the guidelines of Franz Brentano and Edmund Husserl’s methods in their everyday clinical practice and their understanding of mental illness (Broome et al., 2013). The application consisted in obtaining, analyzing, and interpreting descriptions of the conscious experiences uttered by patients in order to evaluate them as symptoms of a defined or alleged mental disease or syndrome. Such an approach constituted innovative and substantial progress in the exploration of other people’s experience and in the development of a systematic mental examination, even though the material of the verbal reports and other expressions of the patients were considered primarily to detect and categorize mental symptoms (Taylor, 1967). The present-day neurophenomenological approach would employ the phenomenological method to the meticulous analysis of verbal reports without a primary psychopathology stance to evaluate any conscious experience. The epistemological requisite would be to treat such verbal expressions as raw data in a sense not very dissimilar to that of videotape recordings of behavior for quantitative ethology and other behavioral sciences requiring further and systematic analyses in order to represent such manifestations in manageable and meaningful transcriptions and models (Díaz, 2007).^2^ The present work proceeds from these considerations in order to explore the possibility of a careful and systematic narrative analysis of first-person subjective reports as indicators of the structure and dynamics of human consciousness.

From a phenomenological standpoint, consciousness can be regarded as a cinematic or narrative stream of explicit mental events. The word consciousness is used here as being analogous to awareness: the acts and processes of an individual while experiencing mental events such as perceiving, feeling, thinking, believing, imagining, remembering, desiring, intending, attending, manipulating, acting, and the like. A scientific analysis of consciousness crucially depends upon empirical methods to analyze such mental phenomena and processing. Since we do not have a direct consciousness meter we must continue to rely upon first-person verbal reports, the traditional manner to access conscious mental acts by the first-person recount and expression of introspection and by the third-person reception and interpretation of such recounts. After the surge, banning, and rebirth of introspective methods during the 20th century, it has been said that verbal reports obtained in controlled conditions and subjected to *inter-subjective* analyses constitute the best tools to study conscious processes and a plausible form of *neomentalism* (Paivio, 1975). Although they can be challenged on several theoretical grounds and there is considerable room for improvement, introspective first-person reports can be so treated as to yield *trans-subjective* data conforming to standard rules of the prevalent scientific method (Díaz, 2007). Moreover, well-structured first-person reports can be used not only as raw data to carry out experiments relevant to neurophenomenology and cognitive neuroscience, but can also be important sources of data for modeling the dynamic structure of consciousness. In order to test such a proposal, it would be necessary to simultaneously work out the discursive forms of suitable first-person reports, a system for transcribing them into phenomenological texts, and techniques to extract from such texts dynamic diagrams and models. Indeed, a narrative approach in consciousness research requires the breakdown of boundaries among cognitive, neural, literary, and philosophical disciplines in order to provide a structured, plausible, and empirically testable explanation of narratives in relation to conscious states and processes (Fireman et al., 2003). A wide field of narrative inquiry emerging in the social sciences already provides theoretical and technical tools in the analysis of discourses and texts (Bloom et al., 1994; Andrews et al., 2008).

In this paper, I will advance some considerations, proposals, and analyses leading to a *narrative* method for the study of first-person reports as expressions of conscious processes.^3^ The general objective of this method is to generate criteria for the production, selection, and transcription of texts that describe subjective experiences and to develop a method of analysis that allows for their interpretation and representation in terms of the structure and dynamics of consciousness, altogether an essential requirement of a systematic neurophenomenology platform. Two main hypotheses of the program are (1) that certain types of narrative that may be called *phenomenological texts* constitute a privileged expression of conscious experience, and (2) that a dynamic-system narrative approach to these expressions allows for the analysis and modeling of the contents and dynamics of conscious processes.

In order to accomplish the task, this paper is divided in sections proceeding from a general picture of first-person narratives to an analysis and plotting of two relevant texts in the shape of a dynamic-system portrayal of mental processing. Thus, in the first section the relevance of introspective language and narratological analyses to consciousness research is explored, and the idea that some narratives are of paramount interest for such investigation is justified. Some of the obstacles to attain plausible consciousness inferences from narrative texts are examined in the second section, and several constraints are described. The most important constraint is the assertion that only a few types of narratives, such as interior monologs, thinking aloud procedures, and some other subjective-state testimonies and discourses, can be used with this aim. The third section examines the methodological requirements of a procedure designed to extract and depict items relevant to consciousness contents and operations from a suitable phenomenological text. In the fourth section a preliminary exercise of the proposed method is used to analyze and chart an excerpt of James Joyce’s *Ulysses* a classic example of the “stream of consciousness” literary technique. Finally, a treatment and evaluation of another phenomenological text, an excerpt from the *Intimate Journal* of Unamuno (1970) using some inter-observer agreement mathematical tools is presented.

## STREAMS OF CONSCIOUSNESS, HETEROPHENOMENOLOGY, AND LITERARY CRITICISM

In chapter 4 of his well-known book *Consciousness Explained*, the philosopher of mind Dennett (1991) put forward the idea of *heterophenomenology*, a program of consciousness research consisting of the extraction and transcription of first-person accounts and their interpretation as a fictional narrative text by trained observers. The heuristic promise of heterophenomenology would take the *protocol analysis* (Ericsson and Simon, 1984) and *empirical phenomenology* (Klein and Westcott, 1994) methods a step further in the direction of modeling consciousness processes and dynamics. In fact, the claims of heterophenomenology have been extended, in both first- and third-person perspectives, to legitimize the inferences of animal consciousness by the concurrent use of physiological, cognitive, and behavioral data (Radner, 1994).

Another way of exploring the promise of heterophenomenology would be to treat introspective verbal reports not merely as *fictions of a sort*,^4^ but as possible genuine renditions, since their narration is an ancient and efficient instrument for recognizing and evaluating actual mental states in others indicating that such narrations communicate several aspects of the form and content of the conscious processes that produced and/or correspond to them. It is by examining diverse types of introspective texts and developing the tools to attain plausible representations and interpretations of these verbal reports in terms of the structure and dynamics of consciousness that the heterophenomenology promise may be put to the test. Not only the human capacity to introspect conscious experience (Goldman, 2006) and the *reportability* of mental processes are indicators of conscious processing (Cohen and Schooler, 1997), but the second and third-person ability to recognize and interpret consciousness states or intentions in the speaker or writer (Hirsh, 1967; Gibbs, 1999) are fundamental tools in interpersonal communication traditionally employed in daily life, in literature, or in the clinical practice of medical semiology, psychopathology, and psychotherapy. The use of autobiographical or soliloquy texts, such as diaries, chronicles, and descriptions or simulations of experience produced by either accomplished or naive writers and speakers, would be a particularly favorable approach (Díaz et al; 1998). This idea has precedents in literary theory and criticism (Cohn, 1978; Pimentel, 2012) some of which may be used to specify the linguistic characteristics of first-person introspective accounts, which in turn may be used to generate approaches that enrich our understanding of the structure of consciousness.

One of the precedents that may be invoked is the venture of French philosopher Bachelard (1982) to study the dynamics of imagination as a basic form of consciousness through the analysis of an immense amount of literary images. His approach was that of a phenomenologist who partakes in the experience of the mental image (Vigneault, 1984). Another relevant case is that of the literary discipline of *narratology*, the formal study of narrative genres, systematics of narrative, and structure of the story (Ryan and Alphen, 1993). Some contemporary trends in narratology are of cognitive interest because they have developed with the general framework of Chomsky’s linguistics and generative grammars. For example, narrative grammars attempting to capture cognitive processes have been integrated into the semantics of narrative action (Schank and Abelson, 1977). Philosophers of the stature of Ricoeur (1985) have also raised the converse issue of the cognitive value of narrative structures. In this context, the Geneva School of phenomenological literary critics have tried to describe and reconstruct the precise ways in which the world is given to the consciousness of both reader and writer through the agency of a narrative (Poulet, 1970). A cognitive approximation to first-person narratives attempts to understand the nature of consciousness by the analysis of oral or written productions of natural language (Chafe, 1990, 1994). From these ideas we may adopt and justify the emphasis of addressing conscious experience as depicted in the text and suspend considerations of cultural, biographical, or historical influences. The narrative method of inquiry should be restricted to the literary language that arguably is the fabric of the writer’s phenomenological consciousness.

It is of course important to realize that consciousness is prominent in all narrative. In fact, it is a specific feature of narrative that the realistic presentation of consciousness of a fictional character can create the illusion of immediacy; that is, the illusion of direct insight into the character’s mental life (Stanzel, 1984, p. 126). The presentation of internal experience in a novel is connected with the *narrative mode* and with *perspective*, the choice of point of view or standpoint from which a story is narrated. Such fundamental features of fictional narrative are also true for subjective experience, ordinarily endowed with an intentional stance and a point of view. A selection of information and reduction of data are also characteristic features of both consciousness and literary narration. The process in a narrative may be at the same time an account of behavioral actions or historical events and a description of the role mental processes of a character play in the generation of those actions and the impact they have upon the character’s inner world. From such a dual setting of narrative, the noted cognitive psychologist Bruner (1986) derives the proposal of two types of thinking, one *paradigmatic*, dealing with physical reality, truth, action, and external observation, and the other *narrative*, having to do with mental experience and with the *intentionality* of mental processes. Moreover, authors of narratives need to draw from folk psychological *paradigm scenarios* to describe how certain states of consciousness, especially emotions, are elicited. For Oatley and Jenkins (1992), such scenarios are widely prevalent as plot or script units (schemata) that are also used in the autobiographical narratives uttered in psychotherapy and constitute a core of the concept of personality. After a careful analysis of the structure and meaning of narrative texts, the sociologist of language Chafe (1990) concludes that narratives provide evidence for the nature of the mind and are important vehicles for mental research and the understanding of consciousness.

Although all works of fiction have a background of *implicit* consciousness that takes shape in a reader’s experience (Valdés, 1982, p. 41), the modern novel also seeks to depict consciousness *explicitly* in the foreground text. Thus, in direct response to Henri Bergson and William James, a post-symbolist literary trend called “stream of consciousness” developed in England and France during the dawn of the 20th century. The movement included some works by Woolf (1925/2012, who wrote its credo: see epigram) and especially *Ulysses* by James Joyce and Marcel Proust’s *A la recherche du temps perdu,* two ground-breaking pillars of the modern novel. Also in William Faulkner’s novels, especially in *As I Lay Dying*, a series of stream-of-consciousness monologs appear without the presence of a narrator. This technique turns the psychology of a character into a dominant concern and is able to present it with much more complexity and authority than the more traditional narrative styles. According to Pope and Singer (1978), this literary trend had lasting consequences for other forms of artistic expression, including the innovative films of Russian director Sergei M. Eisenstein.

In reference to the possibility that these types of texts may be taken as actual descriptions of consciousness, it is necessary to caution that nothing is further from the stream-of-consciousness fiction than direct or automatic writing of what comes to the mind of the author. In fact, except for a thinking aloud or autonomous monolog mode of expression, it would seem quite impossible for writers to keep up with the stream of their own consciousness and its multimodal verbal and non-verbal phenomenological content so as to faithfully capture it in ongoing words. Actually, stream-of-consciousness texts are extremely careful and controlled productions of what an author envisages as taking place in the conscious and subconscious mind of a fictional character. Accordingly, the resulting literary piece is produced by a narrator of an invented and therefore *simulated* consciousness (Steinberg, 1958). To be sure, in order to be able to invent what goes on in the mind of a character authors need to examine the workings of their own mind, to infer the workings of other people’s minds, and in this way to develop and use an implicit “theory” of mind and consciousness.

The modern novel introduced early last century with the masterpieces of James Joyce and Marcel Proust constitutes the most skillfully crafted resemblance of human consciousness available. This can be asserted for several reasons: the implicit theory of mind and consciousness used by each author, the process of consciousness as the author’s principal subject, the extraordinary literary devices used, and the reader translation of the simile into an awareness of a recognizable state of mind are some of the reasons to assert such resemblance. Because of similar deliberations, Ricoeur (1985) considered that no art has gone as far as the novel in the representation of consciousness and proposed that its developed diversity and flexibility have made it a privileged instrument for the investigation of the human psyche. M. M. Bakhtín, another prominent literary scholar of last century, claimed that the novel represents the best example of the *dialogic* character of consciousness with its representation of *alterity* (Radzikhovskii, 1987). Moreover, according to Belgian literary critic Poulet (1970), consciousness is the point at which author and reader converge because the characteristic condition of narratives is that readers summon back a literary work by placing their consciousness at its disposal. Furthermore, the noted novelist and philosopher Murdoch (1992, pp. 258–264) claims that the novel exhibits the ubiquity of the truth-seeking and value-laden moral quality inherent in consciousness. Among many others, she offers the powerful example of the consummate moment towards the end of Proust’s classic *A la recherche du temps perdu* when the narrator experiences a pure state of joy and certainty after stepping and slipping on an uneven paving stone.

A potentially fruitful possibility is to try to infer and analyze the theory of consciousness used by writers to simulate the mental states of their characters, a theory that most likely explains their behavior or further cognition. The existence of a system of causal law-like explanations would imply a *narrative psychology*, a developed variety of *folk psychology* quite worth exploring. Such narratological analysis would be a significant step, provided that it succeeds in showing the structure of consciousness according to a given author. Ascertaining whether an author’s model is correct would be a separate issue initially requiring comparisons with other authors and cognitive science models. Such a narrative research into consciousness structure is strengthened by several broad proposals emerging since the 1990s. Thus, a “marriage” between narratology and cognitive science was proposed in 1994 by Nobel laureate Simon (Simon, 1994), at the same time that qualitative methods of discourse analysis started to develop (Denzing and Lincoln, 1994), and an interdisciplinary field of *narrative psychology* was projected (Hevern, 1997).

From this concise review of a disperse but vigorous research endeavor, it is possible to conclude that over the decades a number of literary critics and narratologists have developed an erudite and refined inquiry of consciousness expression in texts that is of potential value to the cognitive neuroscience of consciousness. Despite the usual and required academic controversies in the field, their attempt has been logically rigorous and for the most part germane and comprehensible. A true interaction or even an inter-discipline between criticism and neuroscience seems probable if an inclusive method to explore consciousness that takes into account both its verbal phenomenological expression and neurobiological aspects is purposefully designed and developed. An ultimate objective of this program would be not only to provide a rigorous method to detect and evaluate consciousness contents in phenomenological texts, but to use this technique to correlate with brain images in a genuine neurophenomenological approach.

## THE INTERIOR OR AUTONOMOUS MONOLOG: A SUITABLE INTROSPECTIVE REPORT

The possibility of inferring the structure of consciousness through the analysis of fiction raises several thorny problems concerning the relationships between literary techniques and actual stream of consciousness features. To begin with, it is necessary to reinforce that we should concentrate on the manifest rather than the latent content of a text because it is the process and the phenomenology of consciousness what is of interest for modeling streams of consciousness. The latent environmental, historical, cultural, or psychoanalytic components—though they are certainly present in all works of fiction and strongly engage the reader’s consciousness—can only be approached from a different stance; for example, that of a hermeneutic interpretation (Valdés, 1982; Ricoeur, 1985). But even if we agree to concentrate on the manifest textual description, in order to take a literary work as an indicator of the operations of consciousness, we would need to surmount a triple barrier because the author makes up a consciousness and fashions it into a literary form that the reader eventually experiences as an invented consciousness in a trans-subjective form. The questions of (1) how an author imagines a flow of consciousness, (2) how he or she manages to simulate and express the verbal and non-verbal subjective processes, and (3) how to extract any consistent information about consciousness through the analysis of fiction would need to be carefully sorted out.

Some literary critics (Humphrey, 1954; Steinberg, 1958) have noted that in order to produce an illusion of uncontrolled narrative flow, the author has recourse not only with words but also with verbal and punctuated disguises of sensations, perceptions, and images of different levels of abstraction. Perhaps such pragmatic means could be systematically defined in order to help encode general consciousness contents. As for the phenomenological engagement of reader and text, it is possible to conceive standard inter-evaluator agreement procedures for encoding and transcribing the text that would render it more objective (in the sense of being inter-subjectively calibrated). I will return to this after dealing with the first two barriers.

In her exhaustive analysis of consciousness modes of presentation, narrative mediation, and point of view in literature, Harvard’s literary critic Cohn (1978) showed that consciousness may be presented in a text in either third- or first-person contexts.^5^ In third-person texts, consciousness may be described in three main ways: (1) *psycho-narration*, narration of what a character is thinking or feeling, (2) *quoted interior monolog*, direct thought quotation, and (3) *narrated monolog*, a character’s mental discourse in the guise of the narrator’s discourse. In first-person texts, consciousness may be described in similar ways: (1) *self-narration*, retrospective description of a mental state, (2) *self-quoted monolog*, quotations of past thoughts, and (3) *self-narrated monolog*, when the narrator identifies with his or her past self.

Cohn further distinguishes such first-person narratives from the *autonomous monolog*, in which the thoughts of the narrator unfold at the moment of locution. This is a variety of present tense discourse previously called *direct interior monolog* and *soliloquy* (Dujardin, 1931/1991; Humphrey, 1954). In this type of discourse narrators describe their actual moods, opinions, memories, and mental circumstances through many literary devices and the prevailing use of the pronoun *I*. In the interior monolog text the *I *abdicates its function of explaining, ordering, selecting, and presenting the elements of the story to the reader, in favor of the direct rendition of the mental processes of the narrator’s or the character’s mind. This is in sharp contrast to the autobiographical novel with its characteristic first-person explanatory narration. The narrating self of the monolog has been substituted by an experiencing self that does not address a reader but embodies a consciousness process in a particular situation (Stanzel, 1984, p. 174). In this way the readers are obliged to place themselves in the character’s position and to experience his or her mind directly in the act of reading. The paradigmatic example used by Cohn to illustrate the autonomous monolog is in “Penelope,” the closing chapter of James Joyce’s *Ulysses*, “the only moment in the novel where a figural voice totally obliterates the authorial narrative voice throughout an entire chapter” (Cohn, 1978, p. 218)*.* As we shall see in a forthcoming section, it is possible to identify verbal renditions of emotions, memories, projects, visual and auditory impressions, or voluntary movements by dividing selected excerpts of this text in syntactic units of meaning and by identifying specific expressive forms and patterns. An interesting variant of the autonomous *monolog* is the *memory monolog *in which a remembered episode plays a privileged role in the present discourse and takes the form of an autobiographical narrative in which the chronological structure of the episode corresponds to the live present of the autonomous monolog so that the string of memories springs up in the monologist’s mind at the moment of locution (Cohn, 1978, pp. 247–250). Clearly, the literary form of the autonomous monolog can accommodate a surprisingly wide variety of mental experiences and, according to Cohn, in ways which affiliate it closely to the “lyric present” of some forms of poetry and drama. Cinema also presents such monologs at times in intense and dramatic renditions. A paradigmatic example of this is Tom’s (played by Henry Fonda) monolog or soliloquy from *The Grapes of Wrath* (Directed by John Ford in 1941):

Well, maybe it’s like Casy says. A fella ain’t got a soul of his own, just a little piece of a big soul—the one big soul that belongs to everybody. Then... then, it don’t matter. I’ll be all around in the dark. I’ll be ever’ where—wherever you can look. Wherever there’s a fight so hungry people can eat, I’ll be there. Wherever there’s a cop beatin’ up a guy, I’ll be there. I’ll be in the way guys yell when they’re mad—I’ll be in the way kids laugh when they’re hungry an’ they know supper’s ready. An’ when the people are eatin’ the stuff they raise, and livin’ in the houses they build—I’ll be there, too.

It seems possible to pose that some thinking aloud (Ericsson and Simon, 1984) and other first-person reports of introspection (e.g., Pope and Singer, 1978, pp. 288–297) used in the cognitive sciences could also be considered narratives in the form of the autonomous monolog. Through successfully bypassing the simulation barrier, these instances can be more readily used to model the structure and operations of consciousness. In order to bypass the simulation barrier, the writer or speaker talk directly from his or her awareness mainly by canceling the intentional stance of communicating to a listener or reader what happens in their mind. Thus, by resorting to a more primitive or *prelogical* mode of thought (Hogenraad and Orianne, 1983), the monologist makes a more emphatic claim to accuracy than if she had used other techniques to depict consciousness. If this inference is correct, and if it is true that these types of narratives retain more veritable traces of experience than others, they can be considered actual data for cognitive neuroscience and neurophenomenology. It seems therefore worthwhile exploring the linguistic and non-linguistic structure and variations of the autonomous or interior monolog and its close relatives in drama and cinema (which add the concurrent domain of non-verbal behavior), as possible forms of expression and analyses of consciousness processes. Given the formidable obstacles of simulation, this literary form could be paramount in a narrative approach to consciousness. Moreover, verbatim recordings and transcriptions of monologs, soliloquies, and other introspective reports produced in medical, psychiatric, and psychotherapeutic settings that intent to directly and accurately express the process and contents of the speakers mental life may constitute the most direct expressions of conscious processes and therefore amenable to cognitive exploration and modeling. Following the ideas of Maurice Merleau-Ponty, the Canadian Education Scholar van Manen (1990) posed that to conduct a first-person narrative or report of an experience is to give a direct description of an ongoing or past experience without trying to embellish, explain, or interpret it.

Even if direct, sincere and forthright descriptions of experience are obtained, it is important to emphasize again that these expressions should not be understood as mimetic or mechanical reproductions of consciousness comparable, for example, to videotapes of behavior as realistic renditions of movements or actions. The monolog would be more comparable to the anecdotal verbal descriptions of behavior that evolved to categorize, identify, sample, and schematize behavior and from which advanced ethological models of behavior have been drawn (Ray and Delprato, 1989).

## A NARRATIVE RECONSTRUCTION OF CONSCIOUS PROCESSES FROM PHENOMENOLOGICAL TEXTS

The modeling of consciousness processes from verbal reports carefully produced and obtained according to the rules of the autonomous monolog or thinking aloud techniques, requires the development of a threefold narrative method focusing on the production and analysis of what has been called a *phenomenological text* (Díaz et al; 1998). The first part of the procedure requires standardized criteria for selecting and/or obtaining a pertinent experience descriptive monolog or a phenomenological text. A phenomenological text is a first-person verbal report that describes states of consciousness by expressing ongoing conscious experience in the present time, that is, in the act of speaking or writing. Phenomenological texts include fiction narratives, which are simulated first-person texts, the mimetic soliloquies, and interior monologs of the “stream of consciousness” literature, and analytic texts or psychological narratives such as Proust’s prose in *A la Recherche du temps perdu*. Phenomenological texts can also be non-fiction factual, candid, and bold first-person texts found in autobiographies, journal excerpts, and psychopharmacology self-experiments. Other types of non-fictional phenomenological texts are transcriptions of introspective accounts such as the factual first-person soliloquies stated in medical and psychotherapy sessions and transcribed in verbatim protocols (Bucci, 1996; Mergenthaler, 1996). A qualitative discourse analysis approach to the verbatim transcriptions in the analytic hour is becoming a motivating research tool to investigate a psychoanalytic process (Siegel et al., 2002). There is, of course, ample room to generate and refine syntactic, pragmatic, and connotation rules for the development of methodical phenomenological texts starting from the stream of consciousness or thinking aloud techniques. Moreover, with continuing practice and correction according to an ever more refined set of rules, human subjects could become expert “monologists” in the production of rich and ever more rigorous phenomenological texts.

The second part of the narrative method requires the development of a system that allows for the identification of items in the phenomenological text that are taken to be indicative of specific consciousness contents and processes. This possibility rests on the argumentation made by Reader’s Response critic Iser (1978)^6^ that, despite the fact that a literary text leads to a wide variety of subjective experiences in the reader, it also contains inter-subjective verifiable instructions for meaning production. In the case of cognitive science, Ericsson and Simon (1984) have led the way with their analysis of verbal protocols, which is based on a precise codification of a list of actions explicitly defined and consistently applied through linguistic processing. In their case the task is more manageable because they usually deal with specific assignments such as problem solving. The prospect of generating a list of mental categories that would be involved in task-independent situations may appear to be staggering and as impossible to attain as a complete ethogram or inventory of behavioral units of a given animal species. Condensed or restricted lists of general mental categories (such as *sensation*, *emotion*, *thought*, *image*, *recall*, *intention*) would be necessary requirements for embarking on such an inquiry. There are methods for analyzing content in verbal samples that can reliably measure some mental and emotional traits that are, in turn, well defined and categorized through the application of standardized classification codes and scales (Gottschalk, 1995).

This narrative method proposes to code phenomenological texts by hand, rather than by computer, even though there are computerized text analysis methods such as linguistic inquiry and word count (LIWC), DICTION, General Inquirer, Differential Language Analysis, and several kinds of sentiment analyses that allow to quickly and reliably detect features of what people say and some subtleties in their linguistic styles. There seems to be of psychological value the detection of particles of speech that include pronouns, articles, prepositions, conjunctives, and auxiliary verbs which serve as markers of emotional state, social identity, and cognitive styles (Pennebaker et al., 2003). In this way, Natural Language Processing has been an active computational research enterprise and recently it has assumed the challenge to detect, analyze, and classify subjective private states, particularly sentiments or emotions under the label of Affective Computing. Even though the objective has largely been focused on opinions, sentiments, and attitudes expressed in social networks as relevant for decision-making processes in marketing and business, their potential application on basic search of consciousness traces in written texts is foreseeable if the obstacles are solved. Montoyo et al. (2012) critically reviewed the field and it is possible to surmise that is rapidly advancing in several directions, but that there remain formidable challenges to meet. In reference to the need to detect and analyze phenomenological texts by hand it seems relevant to mention the following three obstacles of the computational methods in Subjectivity and Sentiment Analysis: (1) to detect specific subjective items and connotations, specially in languages other than English, (2) to interpret the cultural or intentional context of both writer and reader of a given text, and (3) to detect not only the explicit but the implicit expressions of sentiments and many other mental states. For example, the available methods are effective in detecting the polarity orientation of emotional opinions concerning a commercial item or a human personality, but the detection of specific emotions is more difficult except if the names of these emotions are openly depicted in the text, Thus, the main reason to code by hand is that understanding and assessing segments of texts allows for a psychological attribution, scope, accuracy, and depth that still cannot be matched by computerized language measures that are probabilistic methods of word detection, count and use. Even though the results suggests that Affective Computing methods can identify some emotions in written texts, word use is highly contextual and the systems ignore setting, style, irony, sarcasm, meaning, or intent that are paramount features of consciousness and decisive to recognize a person’s state of mind (Tausczik and Pennebaker, 2010). Nevertheless it seems feasible and probably useful to use both computer-based and reader-interpretation systems to increase the reliable detection of specific mental items in phenomenological texts.

An analysis of a phenomenological text would require an *inter-subjective* agreement and calibration among independent evaluators decoding of a text in accord with a set of operational definitions and rules. Such an approach would not attempt or dare to reduce the phenomenon of literature, narrative, or discourse into a discriminating technical procedure, but would consider and use one of its forms, a well-defined phenomenological text, as a linguistic channel for the expression and analysis of conscious experience. The procedure seems more modest and practical than some methods used in literary criticism, such as the phenomenological approach of Valdés (1982) based on textual analysis and inter-subjective (reader–author) interpretation.

Finally, the method would require a procedure for transcribing the coded items into formal devices such as dynamic system diagrams. There has been a growing interest in the development of formal methods for the analysis of narrative texts, some of which seem suitable for the purpose of encoding and transcribing literary, spontaneous, and/or methodical phenomenological texts. Thus, the narrative method of Abell (1993) formalized a finite set of actors, actions, and outcomes in an *algebra* of sequences that are laboriously translated into a “kinematics” graph. Wildgen (1994) proposed a realistic dynamic system analysis of meaning in narrative texts based on an extensive linguistic and mathematical framework that applies image and process schemata to construct pictorial representations. To obtain a graphic representation, narratives are broken into units or episodes, and the information in each one is depicted by vectors representing the function of the main verb of the unit in the two dimensions of text progression and referential content. The aim of the method is to “uncover the metamorphosis of ‘reality’ and lived experience without loosing *the volatile traces of experience*” (Wildgen, 1994, p. 155, emphasis added). These procedures enable the *narrativist* to piece the elements of actions together as parts of a meaningful structure that, in turn, invites interpretation. Nevertheless, the resulting graphical representations are frequently quite abstract and difficult to interpret without extensive information about the coding and transcription criteria.

Since the 1990s I suggested that other dynamic system techniques, such as Petri nets, can be used and may be more intuitively accessible (Díaz, 1997, 2007). Petri nets are important computational models used for the analysis of information processing systems that consist of the explicit graphic representations of the dynamics of a concurrent dynamic system (Peterson, 1981). In Petri nets the process is usually presented by the use of nodes (places and transitions) connected by arcs in which tokens are fired from one place to the next in the transition. Petri nets have been used in the representation of actions (Adorni and Poggi, 1991) that may be relevant to the task of depicting the information coded from a phenomenological text. In 1996 I used an open Petri net diagram to model conscious streams that represented the rising of contents (sensations, emotions, thoughts, images) as nodes or places, the processing as arcs or vectors, and their effects as transitions (arrow tips) in a temporal graph of mental states unfolding from left to right (Díaz, 1996). Such a representation was specifically intended to incorporate elements of phenomenological texts and thereby to model some of the dynamic elements of conscious processes.

The remaining sections of this paper will present two phenomenological texts selected because they fulfill the requirements posed. The two texts are used to apply a procedure of identification of specific conscious contents and processes and, in the first case, to also transcribe the coded items to a Petri net type of graph.

## MOLLY BLOOM’S MONOLOG: A PHENOMENOLOGICAL TEXT ENCODED AS A DYNAMIC SYSTEM

A brief excerpt from “Penelope” in *Ulysses*, previously analyzed by Cohn (1978, pp. 223–225) as an example of autonomous monolog, will be now used to perform a preliminary test of the procedure. Cohn divided the chosen fragment into 30 segments or sentences that are listed below. The words in parenthesis show a tentative encoding of every sentence in linguistic terms and/or possible corresponding states of consciousness in bold characters.

(1) I bet the cat itself is better off than us (*Conjecture*) (**irritation**)
(2) Have we too much blood up in us or what (*Rhetorical question*) (**irritation**)
(3) O patience above is pouring out of me like the sea (*Interjection*) (**despair**)
(4) Anyhow he didn’t make me pregnant as big as he is (*Emphasis*) (**contempt**)
(5) I don’t want to ruin the clean sheets (*Reflection*) (**sensation, wish**)
(6) The clean linen I wore brought it on too (*Reflection*) (**recollection**, **image**)
(7) Damn it damn it (*Interjection*) (**anger**)
(8) And they always want to see a stain on the bed to know youre a virgin for them (*Reflection*) (**image irritation**)
(9) All that’s troubling them (*Same reflection*) (**irritation**)
(10) they’re such fools too (*Same reflection*) (**contempt**)
(11) ) You could be a widow and divorced 40 times over (*Exaggeration*) (**irritation**)
(12) A daub of red ink would do or blackberry juice (*Fantasy*) (**image**)
(13) No that’s too purply (*Correction*) (**unfolding image**)
(14) O Jamesy let me up out of this (*Interjection*) (**tiredness, anxiety**)
(15) Pooh (*Interjection) *(**same**)
(16) Sweets on sin (*Interjection*) (**same**)
(17) Whoever suggested that business for women what between clothes and cooking and children (*Rhetorical question*) (**thought**)
(18) This damned old bed too jingling like the dickens (*Judgment*) (**recollection, sensation**)
(19) I suppose they could hear us away over the other side of the park till I suggested to put the quilt on the floor with the pillow under my bottom (*Reflection*) (**recollection, image**)
(20) I wonder is it nicer in the day (*Question*) (**thought begins**)
(21) I think it is (*Answer*) (**thought progress**)
(22) Easy (*Adjective*) (**thought ends**)
(23) I think I’ll cut all this hair off me there scalding me (*Plans*) (**sensation, fantasy**)
(24) I might look like a young girl (*Judgment*) (**fantasy developing**)
(25) Wouldn’t he get the great sucking the next time he turned up my clothes on me (*Rhetorical question*) (**fantasy**)
(26) I’d give anything to see his face (*Judgment*) (**fantasy ends, wish**)
(27) where’s the chamber gone (*Question*) (**visual image**)
(28) Easy (*Adjective*) (**reassurance**)
(29) I’ve a holy horror of its breaking under me after that old commode (*Judgment*) (**fear, fantasy**)
(30) I wonder was I too heavy sitting on his knee (*Question*) (**fear, recollection**)

This is an excellent example of the type of autonomous monolog that strongly suggests states of consciousness from sensations, perceptions, emotions, recollections, conjectures, fantasies, or purposes and their unfolding. We are under the vivid impression of overhearing a thinking and feeling mind. Note that the expression of conscious processes is conveyed by the expert monologist not only by the meaning of the words chosen, but by linguistic signals detectable as interjections, exclamations, rhetorical questions, or self-referential sentences even in this text devoid of punctuation. Thus, although at some level of analysis the written text must be correlated with ongoing thought, it expresses a much fuller range of consciousness contents than merely thought.

The inference I have made of specific mental states from the discursive and textual elements is tentative and preliminary. In order to codify phenomenological texts more strictly, it would be necessary to secure a list of operationally defined discrete mental states and to submit selected texts to inter observer comparison and strict agreement procedures as will be presented in the next section. In reference to the emotions there has been substantial advance to provide for an encoding technique. Such progress includes a definition of the loci of affect in language (Besnier, 1990), a semantics of emotional word concepts (Wierzbicka, 1992), the emotional value ascribed to words (Campos and González, 1992), and a chromatic model of the affective system developed from the clustering of emotional terms (Díaz and Flores, 2001).

**FIGURE 1:**
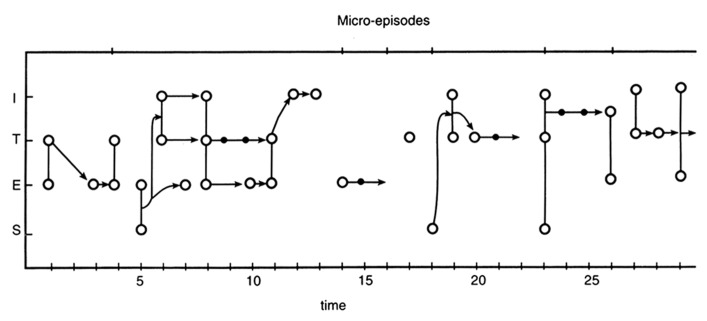
**Flow diagram of linguistic items signaling mental processes encoded from an excerpt of James Joyce’s *Ulysses*.** The phenomenological text is divided in 30 sentence segments (Cohn, 1978, pp. 223–225) and these are plotted as arbitrary units in the temporal dimension of the abscissa. The ordinate shows four general categories of mental contents inferred (see text) from each segment (S, sensations; E, emotions; T, thoughts; I, mental images). Following some rules of Petri nets computational devices (Peterson, 1981) applied in a dynamic process model of consciousness (Díaz, 2007), individual contents detected in each segment are depicted as places (**O**), information processing as arcs (-), and mental causality as arrows (→). Simultaneous mental tokens detected in a single episode are connected by vertical lines to indicate their binding in a single mental state or occasion.

**Figure 1** shows a graph produced following some of the aforementioned Petri net model features of consciousness structure. The encoding of Joyce’s phenomenological text analyzed above is charted in two dimensions. The horizontal dimension of *time* is plotted in accord with the advance of the text in which each segment or sentence is given an arbitrary unitary value. More rigorous temporal criteria could be followed such as the number of letters or words or reading aloud time. But we must consider that the relationships among physical time, clock time, subjective time, discourse time, and diegetic or narrative time is very complex and, indeed, in many ways baffling (Ricoeur, 1985; McInerney, 1991; Díaz, 2011). Four general mental categories are plotted as nodes and vectors in the vertical dimension: (1) sensations and perceptions, (2) emotions, (3) thoughts (including recollections, plans), and (4) mental images (including fantasies). When two simultaneous contents are recognized in a given segment, a vertical line signifies their binding into a single state. Finally, when the linguistic flow and meaning suggest that one mental state is crucially involved in the generation of the next one, such apparent mental causality is depicted by arrow tips located at the end of a vector.

In **Figure 1**, the mental process of the fictional Molly Bloom expressed in the text, can be carefully reconstructed and analyzed in terms of its microstructure dynamics. For example, the process can be segmented into the seven micro-episodes shown in the upper line according to apparent breaks in the causality flow (segments 1–4, 5–13, 14–16, 17, 18–22, 23–26, and 27–30). Each of these segments has a particular subtheme or content process that can be detected in the text. Such particular mental processes can be as short as single items, such as the relatively isolated thought in 17 or emotion in 14–16. They can also appear as a complex amalgam of ensuing multimodal factors and states usually triggered by a sensation and developing around the particular theme (the “stain” sequence 5–13; the “jingling” sequence 18–22; the “cut hair” sequence 23–26; and the “chamber-commode” sequence 27–30). Moreover, the particular themes seem to integrate a larger subject that can be identified in terms of Molly Bloom’s attitudes, history, and personality. The interpretation of these factors would go beyond the task of detecting the microstructure of a consciousness stream. Nevertheless, even in this brief encounter with Molly’s fictional mind we can ratify Steinberg’s notion (1958, p. 232) that we are in the presence of “a shallow mind working over the mulch of everyday existence.”

It is my contention that this model probably depicts parts of the theory of consciousness used by the author in the fabrication of the subjective world of a fictional character. It would be of interest to compare it to other dynamic encoding of phenomenological texts, such as “thinking aloud” transcriptions, journals, dramatic soliloquy, or “lyric present” poems. Such comparison could be indicative of the value of the procedure to model the dynamic structure of consciousness.

## UNAMUNO’S JOURNAL EXCERPT: AN INTER-SUBJECTIVE ATTRIBUTION PROTOCOL

The inter-subjective analysis of a phenomenological text is another empirical avenue to test the hypothesis of the present research proposal and protocol. The ability of research participants to evaluate an introspective journal excerpt in two different tasks was assessed by inter-observer agreement methods. The first task was the division of a selected phenomenological text in thematic segments; the second, the attribution of specific mental category terms to the recognized segments. Sixteen students from the Faculty of Psychology from the University of Querétaro, México, were used as an assembly of judges. They were handed an excerpt from *Intimate Journal* by the well-known Spanish philosopher Unamuno (1970)^7^ and instructed to break the text according to the expression of distinct ideas, not necessarily following grammar or punctuation following the definition of a text segment as a chunk of text that is perceived as a unit. The defined segments appear below with the index of the average inter observer agreement on the right. Since the total average inter observer agreement was 0.60 and this represented a percentage above half of the participant judges, it was decided that any agreement above this level would be significant and serve to establish a segment for further analysis. The asterisk following the index signifies that a partition was established so that, in this way, the text was divided into the following nine segments enlisted below with their English translation below each segment.

**Table:** 

1.	El más insignificante suceso,	0.375
	* The most insignificant occurrence*
	el encuentro de cualquier frase,	0.5
	* any sentence I encounter,*
	la palabramásinocentequeoiga,	0.25
	* the most innocent word I hear,*
	lo que dice mi hijo,	0.625*
	* whatever my son says,*
2.	todo se me antoja aviso y símbolo	0.125
	* everything seems to me a signal, a symbol*
	y cosadesentidooculto,	0.813*
	* and a matter of hidden meaning,*
3.	todo lo traduzco a mi estado.	1.0*
	* I translate everything to my state.*
4.	Si sigo así voy á caer en superstición.	1.0*
	* If I continue like this I will fall into superstition.*
5.	No suenan una vez las campanas	0.063
	* Not a single bell tolls*
	que no crea que me llaman;	0.934*
	* that I fail to believe is calling me;*
6.	se me antoja que se me ha de dirigir á preguntarme que me pasa	0.125
	* I fancy that every man of the cloth that I pass*
	cualquier religioso que veo.	1.0*
	* will approach to inquire what is the matter with me.*
7.	Un deseo grande de declarar mi estado á todos,	0.688*
	* A great desire to declare my state to all,*
8.	una gran felicidad de hacer confesiones a cualquiera,	1.0*
	* a great happiness to make confessions to anyone*,
9.	y una enorme sequedad é indiferencia	0.125
	* and a great dryness and indifference*
	si pienso en hacerla como la Iglesia manda.	1.0*
	* if I think to do it as the Church commands*.

The attribution of mental categories to the nine segments of this phenomenological text was accomplished in the following way. The students were instructed to attribute one or several of the following terms to each segment: *sensation*, *perception*, *emotion*, *thought*, *image*, *recall*, and* intention. *These seven mental process categories were chosen as general classes usually employed in general psychology textbooks, and do not attempt to capture the range of human experience but to include broad-spectrum terms of common use that are encountered both in popular and professional terminology. Depending on whether they judged that the text in the segment expressed one or more of these mental states or categories, the students filled a protocol sheet that was used for statistical analysis. The same seven mental categories were carefully defined in accordance with cognitive psychology criteria in a second experiment. The definitions employed have been offered before (Díaz, 2007, pp. 515–526) but in this occasion I will only present the results for the first test, in which the students were given only the words of these mental functions without a definition. I decided to use only the first results because the aim of this paper is of a more theoretical and general nature. The complete results will be presented in a more factual and experimental manuscript in preparation. The results of the first evaluation appear in **Table 1**.

**Table 1:** Mental term (categories) attribution to a phenomenological text (excerpt from the “Intimate Journal” of Unamuno).^a^

Segment	*n*	Mental categories						
		Se	Pr	Em	Th	Im	Re	In
1	10	0.50	0.60*	**0.30**	**0.30**	0.60*	0.70*	**0.20**
2	14	0.57	**0.21**	0.36	0.36	**0.00**	**0.14**	**0.29**
3	16	**0.13**	**0.19**	**0.25**	0.69*	**0.00**	**0.00**	**0.31**
4	16	**0.13**	**0.06**	**0.06**	0.81*	**0.00**	**0.00**	**0.19**
5	15	**0.27**	0.40****	**0.13**	**0.27**	**0.27**	**0.27**	**0.27**
6	16	0.50	**0.25**	**0.13**	0.37	**0.19**	**0.13**	**0.25**
7	11	**0.27**	**0.00**	0.82*	**0.00**	**0.00**	**0.00**	0.72*
8	16	0.38	**0.06**	1.00*	**0.06**	**0.06**	**0.06**	0.90*
9	16	**0.25**	**0.06**	0.69*	0.82*	**0.19**	**0.06**	**0.25**

a The segments identified by more than half the observers appear in the first column under the legend segment. The number of observers that identified and interpreted the segment is depicted under the letter *n* in the second column. The next seven columns stand for the words that identify particular mental operations in the following way: Se, sensation; Pr, perception; Em, emotion; Th, thought; Im, image; Re, recall; and In, intention. The numbers in the matrix represent the recordings of the category divided by *n*, the number of observers so that 0 means that no evaluator made the attribution and 1.0 that all evaluators made the attribution. Bold numbers are significant at the level of *p* < 0.01 for both high and low scores of the category (Bernoulli hypotheses over proportion test). The asterisk (*) signals the significantly high attribution of the category in the segment.

The significantly high attributions can be interpreted in the sense that this particular mental category was estimated to be present in the segment by this strict inter-observer agreement method. These results indicate high agreement concerning mental activities expressed in the text among this group of students. Indeed, it appears that most of the numbers in **Table 1** are significant both in the mental categories that were felt to be present in the segment, and also in those that were absent. The significantly low attributions indicate that all the judges agreed that those mental categories were *not* expressed in the segment and the high frequency of zeroes means that no observer attributed some mental categories to particular text segments. The opposite case, in which all the observers identified a given mental category to a segment is much scarcer, so that the corresponding number 1.0 appears only once in segment 8 where all the participants attributed an emotion to the segment. In this segment the author actually identifies three particular emotions (happiness [*felicidad*], dryness [*sequedad*], and indifference [*indiferencia*]). Very high levels of agreement were found for the category *intention* for segment 8 (0.90) and *thought* in segments 4 (0.81) and 9 (0.82). In segment 8 the writer speaks about confessing to others, while the intention was more clearly expressed in the previous segment, which also evoked a high agreement for this mental category (0.70). Again, in segment 9 the author uses the verb *to think* (*pienso*). Another interesting result is the significantly high attribution to several categories for single segments, so that about half of them had more than one category (segments 1, 7–9). This result may indicate that a single act or state of consciousness may be a composite of several mental factors in action, one characteristic that has been called the holistic or binding character of consciousness (Díaz, 1996, 1997).

The first segment apparently expresses a perception, an image, and a recollection. The author speaks about having heard sentences in the past and therefore the readers interpret that an actual mental image occurs at the moment of writing because the present tense is used in this memory monolog. It is also interesting that some segments did not receive any significant attribution of mental content, as if it was possible to express an introspective text without content. This occurs in segments 2, 5, and 6 in which most evaluators failed to notice that thinking was necessarily taking place. This particular result shows that undefined mental categories, even if they are given to students of psychology, are not enough to produce reliable inter-observer agreements, beyond the very obvious ones that were already pointed out. Actually in segment 5 the author expresses a belief categorically with the verb *creer*, an evident expression of thinking. As it occurs with untrained observers of behavior, a carefully defined list of behavioral categories is necessary to increase the levels of inter-observer agreement in quantitative ethology.

The present research instrument was shown to have an advantage over a statistical method for examining the agreement of a given population concerning the mental categories attributed to consensual segments of a phenomenological text. Nevertheless, the gap between the writer’s consciousness and the text, and also the gap between the text and the interpretation make for a substantial lag that may be progressively bridged with further refinements of the method up to a certain point. It seems worthwhile to pursue this objective in order to render phenomenological texts more suitable for establishing dynamic models of conscious processes.

## NEUROPHENOMENOLOGY PERSPECTIVES

Taking introspective phenomenological reports seriously in cognitive neuroscience and neurophenomenology research is a fascinating but multiple, challenging, and taxing enterprise. It requires: (1) the development of a theoretical and practical framework to define and choose *genuine* phenomenological reports and texts, (2) a cognitive framework to infer their production and structure in terms of experience, (3) a linguistic method to infer consciousness states and processes from their transcription or written expression, (4) a modeling procedure to quantify, analyze and chart such states, and finally (5) the design of research protocols to correlate the resulting phenomenological items to neuroanatomical and neurophysiological recordings. The ultimate challenge, of course, would be the understanding how and why such psychophysical correlations come about and operate. Nevertheless we have seen that subjective first-person reports expressing conscious processes can be treated so they progressively satisfy methodological cognitive neuroscience and neurophenomenological requirements for relevance, reliability, operational definitions, sampling procedures, inter observer agreement, quantitative analyses, and formal modeling of consciousness. Particularly, it seems that much is to be learned from the application of narrative linguistic–literary analyses and taxonomies to first-person and subjective verbal and written descriptions of the process and contents of consciousness. Also, there is ample room for producing increasingly suitable phenomenological discourses and texts, verbatim renditions and displays, encoding and transcription of indicative elements of such descriptions into formal dynamic-system models. As frequently occurs with novel scientific tools, such Narrative Method for Consciousness Research (*Name-core*) would be initially descriptive and correlative before it could be applied in answering questions concerning the functions of consciousness from the viewpoint of neurophenomenology and the cognitive neurosciences. The method could become a useful tool not only in the understanding of the structure (content and operations) of conscious states and processes but in the analysis and comparison of authors, texts, or evaluators. Moreover, it seems possible to envisage studies of the neural correlates of the verbal and written productions of subjects during the free or increasingly refined expression of their mental states within the stipulations of the autonomous monolog and the narrative method. In order to specify conscious operations and contents, and thereby being able to find their neural correlates, the narrative method would require the stipulation of mental contents by a group of trained evaluators and the retrospective selection of the corresponding periods during their recording by electric or metabolic brain imaging procedures. The brain imaging techniques applied in such a project would require particular adaptations in order to bypass or properly comprise the language production mechanisms that would be common to all conscious states under study.

This article presents a methodology to track consciousness by means of first-person narrations and illustrates the approach by appealing to phenomenological texts, i.e., fiction and non-fiction literary works where vivid expressions of first-person conscious processes can be found. In order to decipher the streams of consciousness that underlie these narrations, this communication presents operational criteria for producing and selecting phenomenological texts, develops an *heterophenomenological* system for detecting conscious contents and processes in such texts, and works out a procedure for representing such items in formal dynamic system devices. Using two example texts, it shows that mental states and processes can be reliably coded in stream-of-consciousness narratives and can be modeled using Petri nets. Finally the paper anticipates a neurophenomenology of consciousness based on the combination of real time recording of phenomenological vocal expressions produced by trained *monologists* during the acquisition of functional brain images. The data provided by a *post hoc* transcription and detection of significantly recognized consciousness contents may be used as a dependent variable of interest to correlate with brain imaging neurophysiological variables.

## Conflict of Interest Statement

The author declares that the research was conducted in the absence of any commercial or financial relationships that could be construed as a potential conflict of interest.

## Footnotes

2 If I understand them correctly, this is what Varela and Shear (1999) mean by mediated verbal reports.

3 The Narrative method for consciousness research, or *Name-core*

4 *Pace* Dennett (1991, p. 81).

5 Pimentel (2012, pp. 117–177) provides an extensive analysis, commentary, and literary examples of Cohn’s categories.

6 Derived from John Searle’s speech–act theory.

7 The original text in Spanish followed by my translation into English: “El más insignificante suceso, el encuentro de cualquier frase, la palabra más inocente que oiga, lo que dice mi hijo, todo se me antoja aviso y símbolo y cosa de sentido oculto, todo lo traduzco a mi estado. Si sigo así voy á caer en superstición. No suenan una vez las campanas que no crea que me llaman; se me antoja que se me ha de dirigir á preguntarme que me pasa cualquier religioso que veo. Un deseo grande de declarar mi estado á todos, una gran felicidad de hacer confesiones a cualquiera, y una enorme sequedad é indiferencia si pienso en hacerla como la Iglesia manda.” “The most insignificant occurrence, any sentence I encounter, the most innocent word I hear, whatever my son says, everything seems to me a signal, a symbol and a matter of hidden meaning, I translate everything to my state. If I continue like this, I will fall into superstition. Not a single bell tolls that I fail to believe is calling me, I fancy that every man of the cloth that I pass will approach to inquire what is the matter with me. A great desire to declare my state to all, a great happiness to make confessions to anyone, and a great dryness and indifference if I think to do it as the Church commands.”
